# *Zingiber officinale* var. *rubrum*: Red Ginger’s Medicinal Uses

**DOI:** 10.3390/molecules27030775

**Published:** 2022-01-25

**Authors:** Shiming Zhang, Xuefang Kou, Hui Zhao, Kit-Kay Mak, Madhu Katyayani Balijepalli, Mallikarjuna Rao Pichika

**Affiliations:** 1 School of Postgraduate Studies, International Medical University, Kuala Lumpur 57000, Malaysia; zhang.shiming@student.imu.edu.my (S.Z.); kitkaymak@imu.edu.my (K.-K.M.); 2 Experimental Centre, Shandong University of Traditional Chinese Medicine, Jinan 250355, China; kouxuefang@126.com; 3 School of Pharmacy, Shandong University of Traditional Chinese Medicine, Jinan 250355, China; zhaohui20222022@163.com; 4 Pharmaceutical Chemistry Department, School of Pharmacy, International Medical University, Kuala Lumpur 57000, Malaysia; 5 Centre for Bioactive Molecules and Drug Delivery, Institute for Research, Development & Innovation (IRDI), International Medical University, Kuala Lumpur 57000, Malaysia; 6 Department of Pharmacology, Faculty of Medicine and Health Sciences, MAHSA University, Selangor 42610, Malaysia; madhu@mahsa.edu.my

**Keywords:** *Zingiber officinale* var. *rubrum*, red ginger, bioactive constituents, biosynthesis, biological activities, molecular mechanisms, pharmacokinetics, analysis

## Abstract

*Zingiber officinale* var. *rubrum* (red ginger) is widely used in traditional medicine in Asia. Unlike other gingers, it is not used as a spice in cuisines. To date, a total of 169 chemical constituents have been reported from red ginger. The constituents include vanilloids, monoterpenes, sesquiterpenes, diterpenes, flavonoids, amino acids, etc. Red ginger has many therapeutic roles in various diseases, including inflammatory diseases, vomiting, rubella, atherosclerosis, tuberculosis, growth disorders, and cancer. Scientific evidence suggests that red ginger exhibits immunomodulatory, antihypertensive, antihyperlipidemic, antihyperuricemic, antimicrobial, and cytotoxic activities. These biological activities are the underlying causes of red ginger’s therapeutic benefits. In addition, there have been few reports on adverse side effects of red ginger. This review aims to provide insights in terms the bioactive constituents and their biosynthesis, biological activities, molecular mechanisms, pharmacokinetics, and qualitative and quantitative analysis of red ginger.

## 1. Introduction

**Figure 1:**
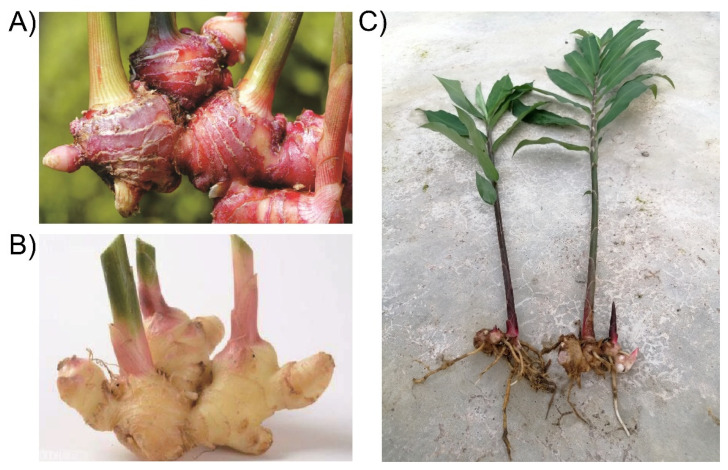
Photographs of (**A**) Red ginger (*Zingiber officinale* var. *rubrum*), (**B**) common ginger, and (**C**) whole plant of *Zingiber officinale* var. *rubrum*.

Ginger, the rhizome of *Zingiber officinale*, consisting of seven species, is mainly distributed in Asia (Figure 1) [1]. Since antiquity, ginger has been used for a wide array of unrelated ailments such as arthritis, rheumatism, sprains, muscular aches, pains, sore throats, cramps, constipation, indigestion, vomiting, hypertension, dementia, fever, infectious diseases, and helminthiasis. The main biological activities of ginger are immunomodulatory, antitumorigenic, anti-inflammatory, antiapoptotic, antihyperglycemic, antilipidemic, and antiemetic. Ginger is a potent antioxidant, and either mitigates or prevents the generation of free radicals. It is considered a safe herbal medicine with only a few side effects.

There is a taxonomic challenge when identifying the correct species as many synonyms are reported for ginger. There are eight plant names for the species *Zingiber officinale* in the plant database (www.theplantlist.org; accessed on 20 November 2021), of which two are accepted names and six are synonyms. Based on the size and color of the rhizome, common ginger can be categorized into three varieties: giant ginger or white ginger (*Zingiber officinale* Rosc. var. *officinale*), small white ginger or emprit ginger Rhizome (*Zingiber. officinale* var. *amarum*), and red ginger (*Zingiber officinale* var. *rubrum*) [1,2,3]. Red ginger belongs to the Spermatophyta division, Angiospermae subdivision, Monocotyledoneae class, Zingiberales order, and Zingiberaceae family. Its scientific botanical name is *Zingiber officinale* Roscoe var. *rubrum*. Its synonyms are *Zingiber officinale* Roscoe var *Sunti* Val., *Zingiber amomum* L., *Zingiber cholmondeleyi* (F.M. Bailey) K. Schum., *Zingiber missionis* Wall., *Zingiber officinale* var. *macrorhizonum* Makino, *Zingiber officinale* var. *rubens* Makino, and *Zingiber sichuanense*. It is red, with a yellow to pink cross section on the outside of the rhizomes, while the base of the leaf shoot is red. It is an annual plant that grows up to 50–100 cm high. The rhizomes are thick and reddish-brown. It is morphologically similar to common ginger. It is smaller and more pungent than common ginger. The leaves are narrow and lancet-shaped, 5–25 cm in length and 8–20 mm in width. The plant has an ovoid-shaped composite that emerges from the rhizomes, with a stem length of 10–25 cm and small leaves at the base of the flower. The corollas are funnel-shaped, 2–2.5 cm long, and dark purple with creamy yellow spots. The petals are small, tubular, and tridentate. Unlike common ginger, its petiole is reddish, and the lip is scarlet red [4]. Red ginger is mainly cultivated in China, Indonesia, and Malaysia. Photographs of red ginger, common ginger, and the whole plant of red ginger are shown in Figure 1 [1].

The Pubmed, Scopus, and Web of Science databases were used to collect the information on red ginger. The keywords used were “*Zingiber officinale* var. *rubrum*”, “Halia Bara”, and “red ginger”. Only articles published in English are included in this review. A few additional references from the web that were deemed useful for the completion of this review article were included.

To the best of our knowledge, this review article is the first on *Zingiber officinale* var. *rubrum*. This review aims to provide an exhaustive summary of the (1) traditional uses, (2) chemical constituents and their biosynthesis, (3) bioactivities and molecular mechanisms, (4) analysis and quality control, and (5) medicinal products. Research gaps in the literature are identified and we have suggested future research opportunities. Perspectives on further improving the medicinal value of red ginger are provided.

## 2. Chemical Constituents of Red Ginger and Its Biosynthesis

The chemical composition of red ginger is complex, containing about 169 chemical constituents [4,5,6,7,8,9,10,11,12,13]. The chemical constituents include monoterpenes, sesquiterpenes, diterpenes, vanilloids, flavonoids, etc. The biological activities of red ginger might be due to the synergistic or additive effects of these compounds. In addition, red ginger contains amino acids, vitamins, and trace elements (iron, copper, manganese, zinc, chromium, nickel, strontium, etc.) [14]. Ghasemzadeh et al. [15] reported that the total number of phenolic and flavonoids in red ginger is higher than in common ginger. Sivasothy et al. [4], using GC-MS, characterized the chemical composition of red ginger oil. It contains three predominant monoterpenes (camphene (14.5%), geranial (14.3%), and geranyl acetate (13.7%)) and 47 sesquiterpenes. Ten flavonoids were reported in red ginger [16]. It has been reported that extraction with carbon dioxide increases the total flavonoid content in red ginger [17]. The major bioactive compounds in red ginger are vanilloids containing a 3-methoxy-4-hydroxyphenyl (vanillyl) moiety [18]. The concentrations of vanilloids are higher in red ginger [19,20] than in common ginger. Based on the chemistry of the side chain in vanilloids, they are divided into gingerols, shogaols, paradols, zingerones, gingerdiones, gingerdiols, etc. [21]. Kusumawati et al. determined the vanilloid content using UV-Visible spectrophotometry [5]. Red ginger’s oleoresin contains 80.06% gingerols and 8.02% shogaols [5]. The vanilloids contribute to the spiciness of the red ginger, of which 6-gingerol and 6-shogaol are the most abundant [18]. Gingerols (4-, 6-, 8-, 10-, and 12-gingerol) are predominant in fresh ginger [22], whereas shogaols (6-, 8-, and 10-shogaol) are predominant in dry ginger [23]. Ghasemzadeh et al. reported optimum extraction conditions to obtain oleoresin with the highest content of 6-gingerol and 6-shogaol [24]. Extraction of red ginger with methanol at 76.9 °C for 3.4 h yields 2.89 mg/g 6-gingerol and 1.85 mg/g 6-shogaol. Eleven vanilloids have been reported in red ginger; the structures are characterized using Nuclear Magnetic Resonance (NMR) spectroscopy and Matrix-assisted laser desorption/ionization-time of flight mass spectrometry (MALDI-TOFMS) [25].

The chemical structures of constituents and their respective compound classes are recorded in Table 1.

### Biosynthesis of Vanilloids

**Figure 2:**
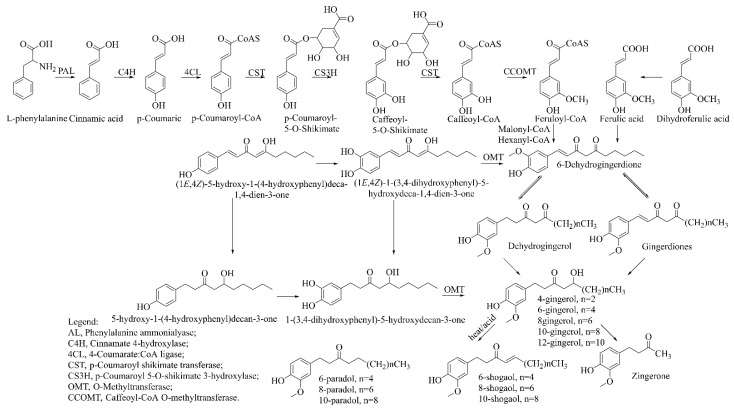
Biosynthetic scheme of ginger pungent compounds [28,29]. The enzymes involved in the biosynthetic pathway to gingerols in ginger are as follows: PAL = Phenylalanine ammonialyase; C4H = cinnamate 4-hydroxylase; 4CL = 4-coumarate: CoA ligase; CST = p-coumaroyl shikimate transferase; CS3H = p-coumaroyl 5-O-shikimate 3-hydroxylase; OMT = O-methyltransferase; CCOMT = caffeoyl-CoA O-methyltransferase.

The vanilloids are biosynthesized from the amino acid phenylalanine, as shown in Figure 2. Shogaols are generated from the dehydration of gingerols’ thermally unstable β-hydroxyl ketone moiety [26,27].

## 3. Biological Activities and Molecular Mechanisms of Red Ginger

Red ginger is reported to possess a wide range of biological and pharmacological activities. In traditional medicine, it is used for treating headaches, indigestion, nausea, vomiting, and cancer. In addition, it is widely used to treat autoimmune diseases [7], hypertension [30], hypercholesteremia [31], hyperuricemia [32], bacterial infections [1], and cancer [1]. A summary of red ginger’s biological activities and molecular mechanisms is given in Table 2.

### 3.1. Antimicrobial Activity

Many studies have reported the antimicrobial and antifungal effects of ethanol and methanol extracts of red ginger [4,64]. Red ginger was found to be safe and, thus, is used as a food preservative [65,66]. Further studies should be carried out to evaluate the molecular mechanisms involved in red ginger’s bacterial and fungal resistance. Philip et al. [33] evaluated the antibacterial activity of red ginger against Gram-positive and Gram-negative bacteria using an agar disc diffusion assay. This study found that red ginger showed potent activity in inhibiting the growth and killing of *Pseudomonas aeruginosa*, *Staphylococcus aureus*, and *Bacillus subtilis*. The rhizome oil was moderately active against *Bacillus licheniformis*, *Bacillus spizizenii*, *Staphylococcus aureus*, and *Escherichia coli* [4].

Red ginger extract has been used as an auxiliary medicine for treating oral infections. Sukandar et al. [64] investigated the antibacterial activity of the combination of red ginger and conventional antimicrobials (amoxicillin, vancomycin, and ketoconazole) against infectious oral microbes. A synergistic interaction between red ginger and conventional antimicrobials was observed against *Candida albicans*, *Staphylococcus aureus*, and *Streptococcus mutans*; therefore, the combinations may help treat orals infections. Another study found that the combination of nisin and red ginger essential oil had a synergistic effect against *Bacillus cereus* [66]. Studies have shown that red ginger can be used as a preservative for milk and milkfish [65]. Irawan et al. [67] have incorporated red ginger in chitosan films as packaging materials for storing milkfish to increase the shelf life. Rialita et al. [68] optimized antimicrobial products containing red ginger. Red ginger essential oil and Arabic gum in a ratio of 1:3 (v/w) showed the best microcapsule characteristics.

The primary antimicrobial compounds in red ginger are monoterpenes, of which *β*-caryophyllene has predominant antimicrobial activity [4]. The major components of red ginger essential oil showing antimicrobial activity were ar-curcumene, zingiberen, β-bisabolene, β-sesquiphellandrene, and camphene [68]. The antimicrobial activity of red ginger essential oil against microbes was in the order *Bacillus cereus* > *Escherichia coli* > *Salmonella typhimurium* > *Pseudomonas aeruginosa* [66] and *Staphylococcus aureus* > *Escherichia coli* > *Aspergillus niger* > *Bacillus cereus* > *Pseudomonas fluorescens* > *Salmonella typhimurium*. In contrast, the fresh red ginger extract showed stronger activity against *Staphylococcus aureus* and *E. coli* [64]. The antibacterial activity of the red ginger extract against *Streptococcus mutans* is more potent than that of common ginger [69]. Many studies have confirmed the stronger antimicrobial activity against a wide range of bacteria and fungi; thus, it is postulated that it can be used as a natural preservative in the food industry.

### 3.2. Analgesic and Antihyperalgesic Activity

The analgesic activity of red ginger is comparable to that of aspirin [38]. Neuropathic (nerve) pain is caused by damage, dysfunction, or injury of nerves. It affects the patient’s quality of life because of its chronicity and intensity. Traditional medicines containing red ginger are used to treat neuropathic pain [70]. Fajrin et al. [36] reported that red ginger oil (200 mg/kg B.W. and 400 mg/kg B.W.) prolonged the latency time toward the thermal stimulus in male mice, demonstrating antihyperalgesic activity. The activity is mediated via inducing gamma-Aminobutyric acid (GABA) action. GABA balances the action of excitatory and inhibitory neurotransmitters in the central nervous system [71]. GABA suppresses glutamate release and blocks intracellular calcium intake, leading to decreased NR_2_B activity and pain sensitization [36]. Another mechanism by which red ginger oil exhibits antihyperalgesic activity is the inhibition of prostaglandin synthesis [72,73]. Red ginger oil could also reduce paw thickness via this pathway. However, red ginger did not reverse paw thickness in a CFA-induced inflammation model, suggesting that a complex mechanism might be involved. Fajrin et al. also demonstrated the antihyperalgesic activity of red ginger in diabetic neuropathy, mediated via strengthening the spinal cord [35]. Camphene and cineole were found to possess analgesic activity via reducing the production of reactive oxygen species (ROS), and thus offered spinal cord protection [34,35].

### 3.3. Antidiabetic Activity

Red ginger inhibits saccharide hydrolyzing enzymes and, thus, can be used for controlling hyperglycemia in type 2 diabetic patients. Safithri et al. [74] found that a drink composed of red ginger and areca nut had antidiabetic activity in vitro. A formulation containing red betel leaves (42%), cinnamon bark (28%), red ginger (15%), and lime (15%) had the highest antidiabetic activity. Deddy et al. used aqueous extracts of red ginger, at a dose of 3 g per day, to treat type 2 diabetic patients [75].

Oboh et al. [37] reported the α-amylase and α-glucosidase inhibitory activities of red ginger. Thus, it is postulated that red ginger can be used as a dietary intervention to manage postprandial hyperglycemia in type-2 diabetic patients; however, detailed studies are yet to be done. Vanilloids have been reported to protect liver function in diabetes [76].

### 3.4. Anti-Inflammatory Activity

Many studies have confirmed that chronic inflammation is the underlying cause for many diseases such as allergies, atherosclerosis, cancer, diabetes, infection, obesity, and neurodegeneration [77,78]. Red ginger inhibits the synthesis of inflammatory mediators (prostaglandins, cytokines, chemokines, and leukotrienes) via inhibiting the expression of cyclooxygenase (COX)-1, COX-2, and 5-lipoxygenase (5-LO) enzymes [39,40,41]. In addition, red ginger is reported to inhibit nitric oxide production by inhibiting inducible nitric oxide synthase (iNOS) expression, which is mediated by the attenuation of nuclear factor kappa B (NF-κB)- [38,41]. 6-gingerol inhibits 12-O-tetradecanoyl phorbol acetate 13 (TPA)-induced ornithine decarboxylase (ODC) activity and inflammation [41]. Sang et al. reported that the anti-inflammatory activity of 6-shogaol is more potent than 6-gingerol [42].

### 3.5. Antioxidant and Free Radical Scavenging Activity

Many studies have confirmed red ginger’s free radical scavenging activity in different test models [43,44,45]. The antioxidant activity of red ginger is correlated with the total phenolic content. The antioxidant activity of the phenolic compounds in red ginger is due to their capacity of donating either electrons or hydrogens to free radicals [79]. Jayanudin et al. [43] have developed chitosan microcapsules containing red ginger oleoresin. Ghasemzadeh et al. [45] reported that freeze-dried red ginger has more free radical scavenging activity than vacuum- or oven-dried red ginger. The antioxidant activity is attributed to the presence of flavonoids and vanilloids. Red ginger strongly inhibits NO production in LPS-stimulated J774.1 cells via inhibiting iNOS [80,81]. In addition to scavenging free radicals, red ginger scavenges superoxide and hydroxyl radicals. It also suppresses xanthine oxidase activity, which generates oxygen-containing free radicals. Red ginger also possesses a strong metal-binding capacity to inhibit lipid peroxidation and AAPH-induced DNA damage [19,80]. It is also reported to inhibit N-formyl-methionyl-leucyl-phenylalanine (f-MLP)-induced ROS production in human polymorphonuclear neutrophils (PMN) [19,81].

### 3.6. Anticancer and Antitumor Activity

**Figure 3:**
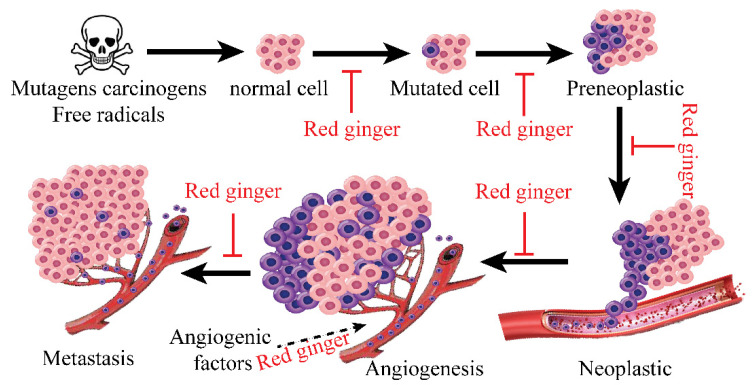
Red ginger inhibits cancer progression, angiogenesis, and metastasis [88].

**Figure 4:**
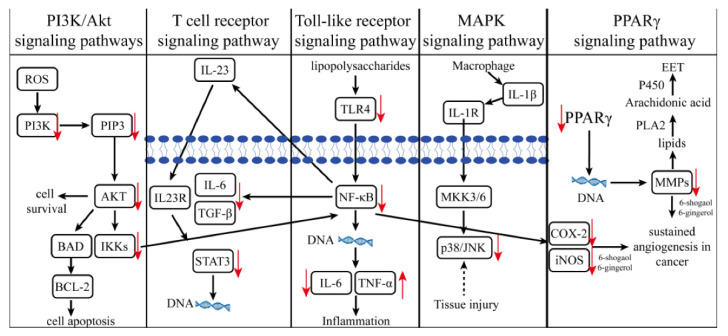
The molecular mechanisms involved in anticancer activity (↓ = downregulation/inhibition, ↑ = upregulation).

The anticancer activity of red ginger is mainly due to certain pungent vanilloids [24]. Several mechanisms have been proposed for the anticancer activity of red ginger [82,83,84]. Response surface methodology was used to optimize the extraction conditions for achieving the highest anticancer activity [24]. Tatsuzaki et al. reported that the highest anticancer activity was observed against human A549, SKOV-3, SK-MEL-2, and HCT15 cancer cells [48]. Dehydrozingerone and its analogues in red ginger exhibited significant cell proliferation inhibitory activity against KB and A549 cells [85]. 6-gingerol inhibited the proliferation of transgenic mouse ovarian cancer cell lines, C1 (p53 (-/-), c-myc, K-ras gene), and C2 (p53 (-/-), c-myc, Akt). The acetoxy, alkyl, and α-β-unsaturated carbonyl groups in the side chain and *ortho*-dihydroxy group on the aromatic ring in vanilloids play a significant role in the anticancer activity [86]. Vimala et al. [87] reported that red ginger inhibits Epstein–Barr virus early antigen activity in Raji cells, suggesting a role of red ginger in preventing cancer in an early stage of progression. Angiogenesis is a key process in tumor migration (Figure 3), and red ginger is reported to inhibit endothelial cell angiogenesis [46,47]. Studies have also shown that 6-gingerol inhibits VEGF- and bFGF-induced cell proliferation, arresting the cell cycle in G1 (by inhibiting human endothelial cyclin D1), which blocks endothelial cells from VEGF [47]. Baliga et al. [88] reported that red ginger inhibits lung metastasis in mice (Figure 3) via antiangiogenic activity and the stimulation of host immunity. These results suggest that red ginger could selectively inhibit the angiogenesis, adhesion, metastasis, and production of MMPs to block the migration of malignant tumors [89]. Multiple mechanisms involved in the anticancer activity of red ginger and vanilloids have been reported elsewhere, including the inhibition of MAPK and PI3K/Akt pathways, inactivation of NF-κB and STAT3, and upregulation of plasminogen activator inhibitor-1 (PAI-1), all of which are participants in the suppression of tumor metastasis [49,50,51] (Figure 4).

### 3.7. Antihyperlipidemic, Antihypertensive, and Antihypercholesterolemic Activity

**Figure 5:**
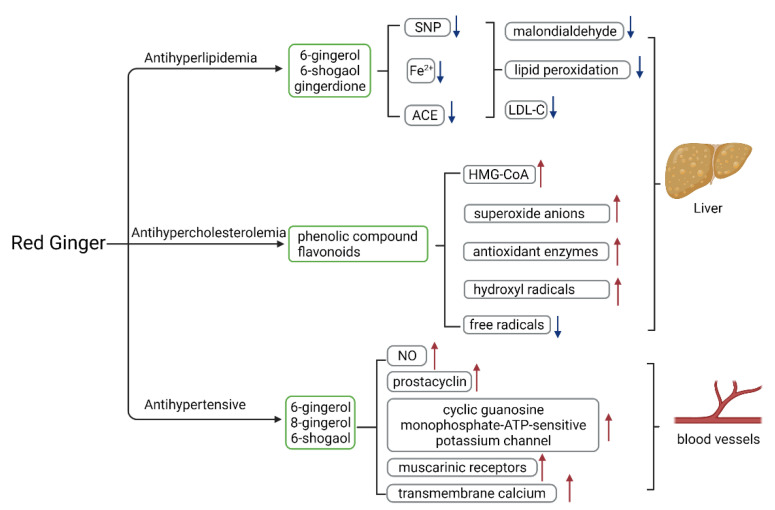
The mechanism of action or red ginger in antihyperlipidemic, antihypertensive, and antihypercholesterolemic activities.

The antihyperlipidemic, antihypertensive, and antihypercholesterolemic mechanisms of action are as shown in Figure 5. Red ginger oil (3.2 mL/kg for 21 days) reduced low-density lipoprotein cholesterol (LDL-C) levels by 12%. The antihyperlipidemic activity of red ginger is attributed to vanilloids and is more potent than that of common ginger [52]. In vitro studies have shown that red ginger aqueous extract (1:20 *w*/*v*) inhibited the actions of Angiotensin-I converting enzyme (ACE), iron(II) ion, and sodium nitroprusside (SNP)-induced lipid peroxidation in rats’ hearts via reducing malondialdehyde levels. The activity of red ginger in this respect is more remarkable than that of common ginger [90]. The antihypertensive activity of red ginger was also demonstrated in cholesterol-fed rats. Red ginger has been reported to reduce malondialdehyde levels in the liver and heart tissues, suggesting that the antihypertensive activity is mediated via inhibiting ACE and lipid peroxidation [52]. Razali et al. [30] demonstrated the antihypertensive activity of red ginger in a SHR (spontaneously hypertensive rat) model. The mechanisms of vascular relaxation involve the release of nitric oxide and prostacyclin, the activation of cGMP-KATP channels, the stimulation of muscarinic receptors, and the stimulation of transmembrane calcium channel or Ca^2+^ release from intracellular stores. The vanilloids are attributed to the antihypertensive activity of red ginger.

It is well known that red ginger extract contains phenolic compounds, which have been shown to protect against metabolic disorders such as hypercholesterolemia. Hypercholesterolemia results from increased cholesterol levels and increased production of free radicals. Phenolic compounds reduce the risk of hypercholesterolemia by (1) increasing the activity of antioxidant enzymes, (2) decreasing the formation of free radicals, hydroxyl radicals, and superoxide anions, (3) inhibiting lipid peroxidation, and (4) regulating low-density lipoprotein (LDL) receptors [54].

Red ginger is reported to (1) inhibit lipid peroxidation, (2) increase antioxidant enzymes, (3) regulate low-density lipoprotein (LDL) receptors, and (4) regulate 3-hydroxy-3-methylglutaryl coenzyme-A receptors (HMG-CoA), which affect cholesterol absorption in the liver [31,91].

### 3.8. Neuroprotective Effect

Oboh et al. have reported the acetylcholinesterase (AChE) inhibitory effect of red ginger in SNP-induced mice [53]. Red ginger has also been reported to protect IMR32 human neuroblastoma, HUVEC cells, and PC12 rat pheochromocytoma cells from amyloid-beta (Aβ) insult [55,56]. Amyloid-beta insult is the major cause of Alzheimer’s disease. In addition, red ginger also significantly improved cognitive and behavioral impairment and AD-like pathology in mice. These beneficial effects occurred via an increase in α-secretase activity and a decrease in cerebral Aβ-42, β-secretase, APH1a activity, and COX-2-linked neuro-inflammation [57]. 6-Shogoal was the most potent bioactive compound responsible for the neuroprotective, neurotrophic, and anti-inflammatory effects of red ginger [58]. The HDAC inhibitory activity of 6-shogaol is comparable to that of Trichostatin A and MS275. 6-shogaol also significantly attenuated various neuroinflammatory responses by inducing HSP70, which is associated with the inhibition of HDAC in cortical astrocytes [20,92].

### 3.9. Androgenic Effect

The vanilloids of red ginger have a potent antioxidant effect [59]. Yang et al. [60] reported that antioxidants could preserve spermatogenesis from the detrimental effects of oxidants. Kanedi et al. [61] also reported that an oral concoction of red ginger extract increases preleptotene and pachytene spermatocytes and spermatids. These studies suggest the androgenic effect of red ginger and its ability to improve sperm quality.

### 3.10. Insecticidal Activity

Mahardika et al. [62] reported the insecticidal and larvicidal activity of red ginger’s hexane extract against *Aedes albopictus*, *Aedes aegypti*, and *Culex quinquefasciatus*. The major principal constituents in the extract were zingerone (14.92%) and benzaldehyde dimethyl thiol acetal (11.61%). Another study reported the insecticidal activity of red ginger against *Spodoptera frugiperda* [93] via increasing cysteine protease’s enzyme activity.

### 3.11. Immunomodulatory Activity

Red ginger is usually one of the ingredients in immunomodulating supplements. A study showed that the administration of black cincau and red ginger had immunomodulatory effects in infected mice. Exposure of infected mice to red ginger and black cincau can help them to recover the small intestine mucosa structure. This study demonstrates that red ginger extract can have an immunomodulatory effect in *Escherichia coli*-infected mice [63]. The combination of black cincau, pandan leaves, and red ginger provides a synergic effect, as shown by the increased levels of phenol and antioxidant activity. The combination of black cincau, pandan leaves, and red ginger provides a synergic effect, shown by the increased levels of phenol and antioxidant activity [63]. Due to the presence of phenol in the red ginger supplement, the condition of mice is approaching recovery. A histopathology examination of the small intestine showed that red ginger can improve cells damaged by an intraperitoneal injection of *E. coli* strain O157 [63].

Red ginger is one of the ingredients in Wedang uwuh (WU, a traditional drink in Southeast Asia). WU has antioxidant activity, can promote blood circulation, and improves immunity. WU extract administered via 27 mL/kg injections had a dose-dependent immunomodulatory potential in diabetic rats. The results showed that WU significantly inhibited the expression of pro-inflammatory cytokines and anti-inflammatory cytokines and achieved a balance between pro-inflammatory and anti-inflammatory cytokines that was not significantly different from that of normal controls. This study’s results confirm that the use of WU can result in immunomodulatory activity in diabetic rats.

### 3.12. Melanogenesis Inhibitory Activity

Yamauchi et al. [25] reported that the melanogenesis inhibitory activity of red ginger and the activity is due to vanilloids. The activity of gingerols is different from that of shogaols because of the differences in permeability into the cell membranes. The glycosylation of vanilloids improved the activity. There were no reports of the mechanism involved.

## 4. Analysis and Quality Control

### 4.1. The Application of Analytical Methods for Assessing Red Ginger’s Quality

Growth conditions influence the chemistry and content of active ingredients in any plant; therefore, developing analytical methods to determine the identity and quality is essential[6]. Two analytical methods, high-performance liquid chromatography coupled with time of flight mass spectrometer (HPLC-TOF-MS) and gas chromatography with a mass spectrometer (GC-MS), have been used for the analysis of red ginger [6,68]. The essential oil was analyzed using GC-MS. The operational conditions were as follows: helium as a carrier gas, a 5% HP-5MS phenyl methyl Silox column (30 m × 250 μm × 0.25 μm), column temperature set to 50 °C, pressure of 7.0699 psi, flow rate of 1 mL/min, velocity of 36.262 cm/s, column flow of 1 mL/minute, split ratio of 25:1, and split inlet temperature of 250 °C. The major components of essential oil are monoterpenes, sesquiterpenes, alcohols, aldehydes, and organic acids. Monoterpenes and sesquiterpenes have strong antimicrobial activity [4]. A total of 54 compounds in fresh red ginger essential oil were reported, out of which the major components are Z-citral (23.332%), citral (18.87%), 1,8-cineole (12.18%), camphene (11.87%), geranyl acetate (3.82%), linalool (2.88%), α-pinene (2.6%), 5-hepten-2-one, 6-methyl-(2.32%), ar-curcumene (2.06%), and α-terpineol (2.05%) [66]. In another study, Nissa et al. reported 20 compounds, of which 81.9% were monoterpenes, and geranial (11.97%) and 1.8-cineole (15.10%) were predominant. The predominant sesquiterpene is αr-curcumene (16.86%). The other components had concentrations of less than 10% [9]. The difference in the essential oil composition could be due to variations in climate, soil, growth conditions, harvest time, and the rhizomes’ age [9]. The major components in essential oil were found to be α-zingiberene, α-curcumene, β-bisabolene, and β-sesquiphellandrene.

### 4.2. Chemical Fingerprint Analysis

The chemical fingerprint analysis was reported using HPLC-TOF-MS[6]. The optimum chromatographic conditions were: source voltage, +4.5 kV (positive ion mode) or −3.5 kV (negative ion mode); capillary temperature, 200 °C; and nebulizer gas flow rate, 1.5 mL/min. The mass spectrometer was operated in positive and negative ion modes, scanning from 150 to 1500 *m*/*z*. A Waters Atlantis T3 column (2.1 mm × 150 mm, 5 μm) was used and the column temperature was maintained at 40 °C. The mobile phase was a binary eluent of (A) 5 mM (NH_4_)OAc solution and (B) CH_3_CN under the following gradient conditions: 0–30 min, linear gradient from 10% to 100% B; 30–40 min, isocratic at 100% B. The flow rate was 0.2 mL/min.

## 5. Medicinal Products

Red ginger has gained traction, especially in Asia, where it is known for its medicinal effects: dispelling wind from the body, relieving indigestion, improving blood circulation, and relieving inflammation. The beneficial effects attributed to the constituents of red ginger have occasioned the creation of various medicinal products. In Southeast Asia, red ginger is locally known as halia bara, halia merah, or jahe merah. Currently, the red ginger products marketed in Southeast Asia include foodstuffs such as dried ginger pieces and pickled ginger, and red ginger extracts in instant beverages such as tea and coffee. Furthermore, red ginger extracts are also added to body lotions, creams, ointments, and capsules.

## 6. Our Perspectives

Our research group has avidly studied ginger, its constituents, and their pharmacological properties. We prepared this review to present a concise summary of the information on red ginger gathered from the scientific literature thus far. We believe that this will be useful to researchers who are working on ginger, natural products, functional foods, and even ethnomedicine. From our perspective, based on our experience with ginger variants, the following are the main differences between red ginger and common ginger. They are: (1) they differ in color and size; (2) fresh red ginger is more pungent than fresh common ginger; (3) upon drying, red ginger turns black, while common ginger does not; (4) dried red ginger is much harder than dried common ginger; (5) the constituents are reported to be the same in both gingers; however, red ginger is reported to be more biologically active; (6) a few studies have reported that the active constituents (gingerols and shogaols) are higher in red ginger than in common ginger; (7) it is very difficult to distinguish these two variants based on the chemical composition via simple analytical techniques using HPLC and GC instruments; (8) there have been no studies characterizing the constituent that gives red ginger its color; (9) there are no studies on why red ginger is hotter than common ginger; and (10) there are no detailed studies that compare the bioactivities of red ginger and common ginger under the same experimental conditions that clearly explain why red ginger is preferred in ethnomedicine, etc.

To date, conclusions on the differences between red ginger and common ginger have yet to be drawn. We strongly believe that it is still worth scientifically exploring the effects of red ginger. The research should be carried out on a standardized extract rather than individual compounds in the physiologically relevant models and molecular mechanisms. It is well established that vanilloids are unique compounds in all ginger variants. Based on our experience with ginger, we believe that red ginger’s chemistry (both primary and secondary metabolites) is different from common ginger, giving rise to a difference in the hotness and biological activities. In our opinion, the plant preparations (consisting of active and inactive constituents) in ethnomedicine are equivalent to modern formulations (consisting of active pharmaceutical ingredients (API) and excipients). It is well known that excipients in a formulation influence the bioactivity of API. Similarly, the inactive constituents in a plant extract also influence the bioactivity of the active constituents.

Red ginger extracts are scientifically validated for their beneficial effects in inflammatory diseases, metabolic diseases, cancer, neurological diseases, and cardiovascular diseases. Research in these disease areas is still a priority for many researchers, institutions, and the pharmaceutical industry. Thus, like herbal medicine, we argue that red ginger has a lot of potential to provide breakthroughs for these diseases. The ginger extract, ginger oil, and zingerone (pungent components of ginger) are included in DrugBank (https://go.drugbank.com/drugs, accessed on 20 November 2021), which indicates the potential for ginger to be used in modern medicine for therapeutic purposes.

## 7. Conclusions

This review summarizes the latest research progress on red ginger regarding its chemistry, ethnomedicinal uses, biological activities, molecular mechanism, and analytical methods. We have provided our perspectives on the value of red ginger in metabolic and neurological diseases. The vanilloids are unique to ginger, and are stable in ginger. However, upon isolation in pure form, they are not stable at room temperature. Although vanilloids are simple molecules, their synthesis is still challenging. Thus, further exploring the potential of red ginger in medicine is essential. Many herbal formulations containing red ginger are famous in various traditional systems of medicine. Many scientific studies worldwide have proven the efficacy of red ginger in a wide array of diseases—metabolic, neurological, cardiovascular, infectious, and cancer. Despite red ginger being relatively safe [94,95], consuming large amounts of ginger can cause hypoglycemia and spontaneous miscarriage [96,97]. However, there are no reports of clinical studies om red ginger.

## Figures and Tables

**Table 1:** The main chemical constituents of red ginger.

Name	Chemical Structure
Vanilloids	
6-Gingerol	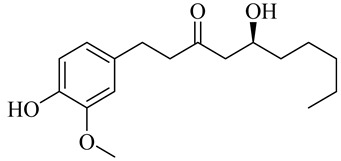
8-Gingerol	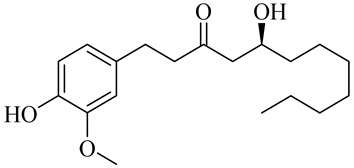
10-Gingerol	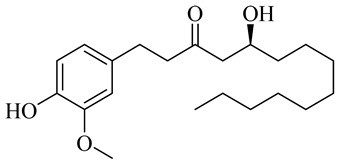
6-Shogaol	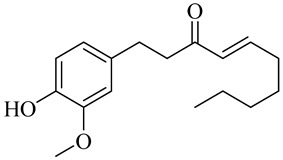
8-Shogaol	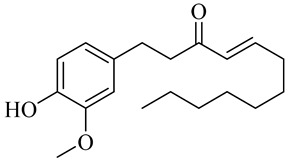
10-Shogaol	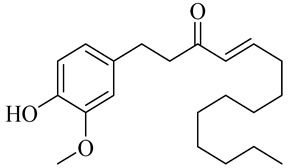
(3S,5S)-[6]-Gingerdiol	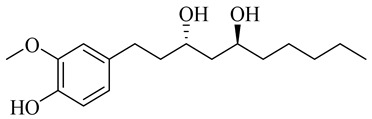
[6]-Gingerdiol 3S,5S-diacetate	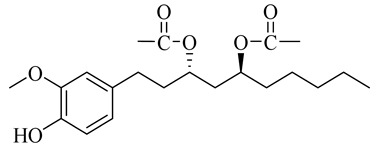
(3R,5S)-[6]-Gingerdiol	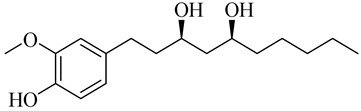
[6]-Gingerdiol 3R,5S-diacetate	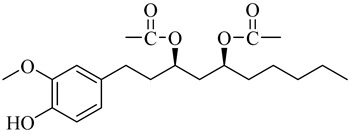
(3S,5S)-[8]-Gingerdiol	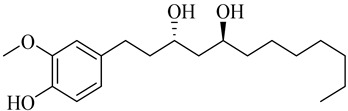
[8]-Gingerdiol 3S,5S-diacetate	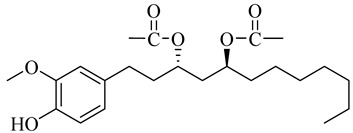
(3R,5S)-[8]-Gingerdiol	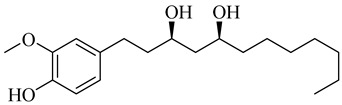
[8]-Gingerdiol 3R,5S-diacetate	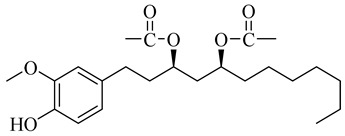
(3S,5S)-[10]-Gingerdiol	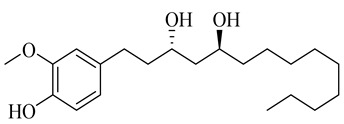
[10]-Gingerdiol 3S,5S-diacetate	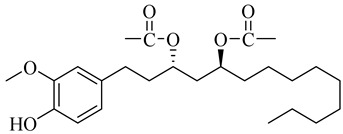
(3R,5S)-[10]-Gingerdiol	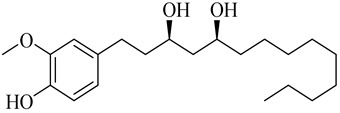
[10]-Gingerdiol 3R,5S-diacetate	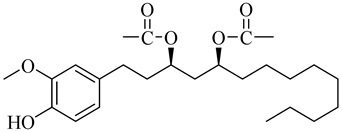
5-Methoxy-1-(4-hydroxy-3-methoxyphenyl)-3-decanone	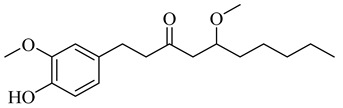
5-Methoxy-1-(4-hydroxy-3-methoxyphenyl)-3-tetradecanone:	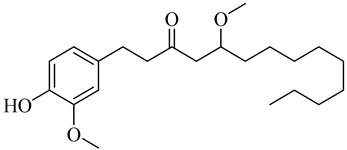
1-(4-Hydroxy-3-methoxyphenyl)-3,5-decanediol	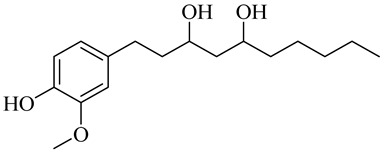
4-Gingeracetate	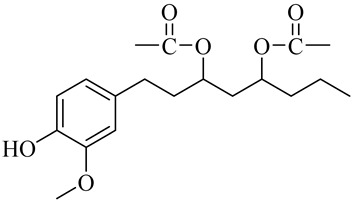
3-Hydroxy-1-(4-hydroxy-3-methoxyphenyl)-5-decanone	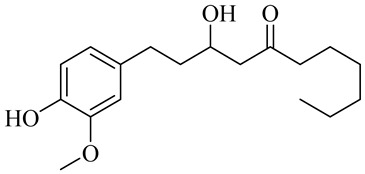
3,5-Diacetoxy-1-(4-hydroxy-3-methoxyphenyl)decane	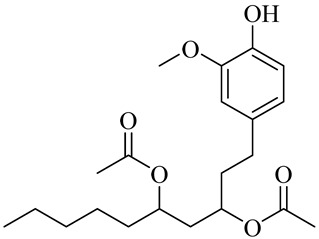
1-Dehydro-6-gingerdione	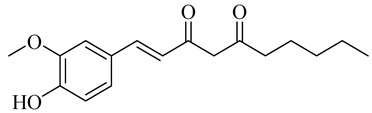
6-Dehydro-gingerdione	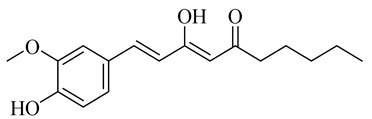
Hexahydro-curcumin	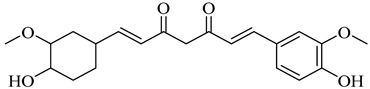
Monoterpenes	
Tricyclene	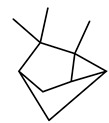
α-Pinene	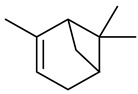
Camphene	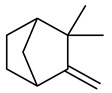
Sabinene	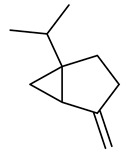
β-pinene	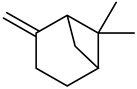
α-Phellandrene	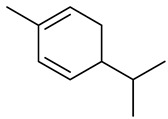
p-Cymene	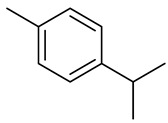
β-Phellandrene	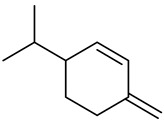
γ-Terpinene	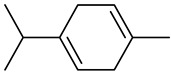
Terpinolene	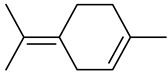
Linalool	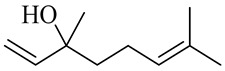
trans-3(10)-Caren-2-ol	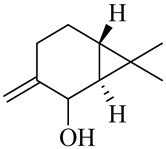
1,1-Dimethyl-3-methylene2-vinylcyclohexane	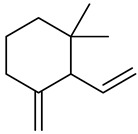
2-Methoxy-1,7,7-trimethylbicyclo [2,2,1] heptane	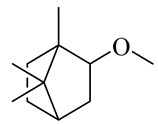
Citronellal	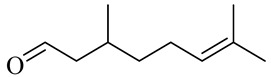
Camphor	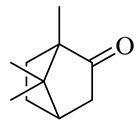
Borneol/Isoborneol	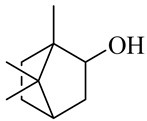
1-Terpinen-4-ol	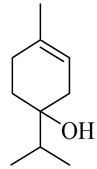
α-Terpineol	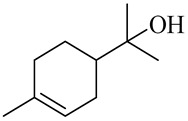
Myrtenal	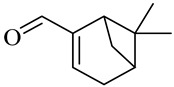
Citronellol	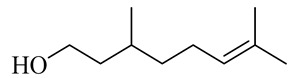
Neral/Z-citral	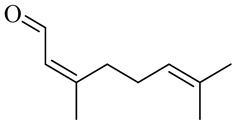
citral	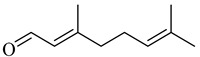
trans-Geraniol	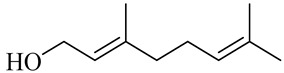
cis-p-Menth-2,8-dienol	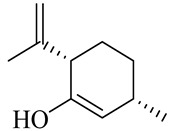
Geranial	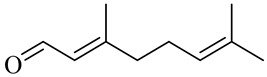
Cyclosativene	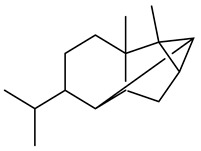
Eucalyptol/1,8-Cineole	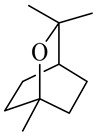
Geranyl acetate	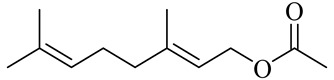
Bornyl acetate	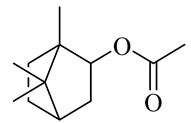
Myrcene	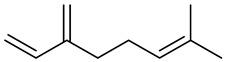
Dipentene/Limonene	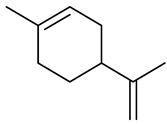
Neryl acetate	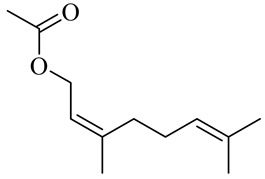
Camphene hydrate	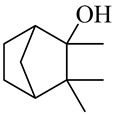
Pulegone	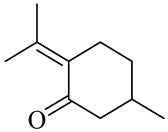
Citronellyl acetate	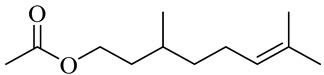
trans-nerolidol	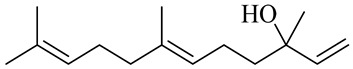
(-)-δ-3-carene	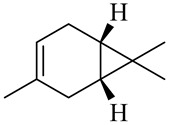
cis-β-Ocimene	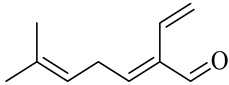
trans-Sabinene hydrate	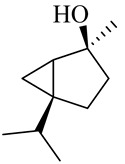
Linalyl formate	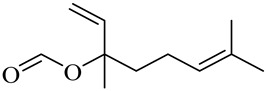
Myrtenyl acetate	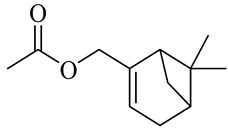
Sesquiterpenes	
α-Copaene	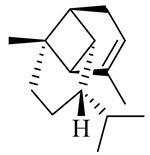
β-Elemene	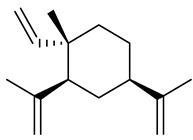
α-trans-Bergamotene	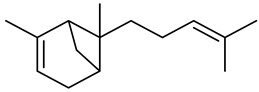
β-Cubebene	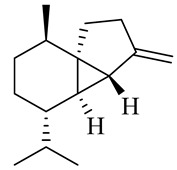
β-Farnesene	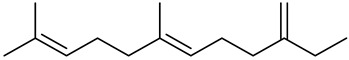
Cedrene	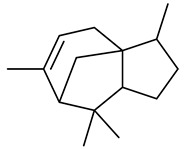
Allo-aromadendrene	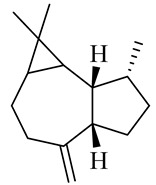
β-Himachalene	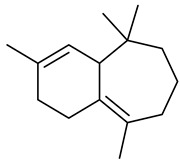
α-Curcumene	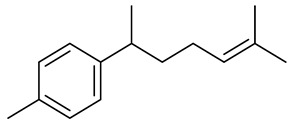
Zingiberene	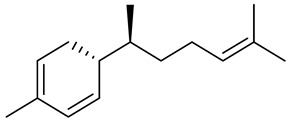
α-Farnesene	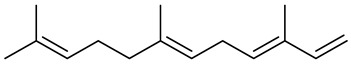
β-Bisabolene	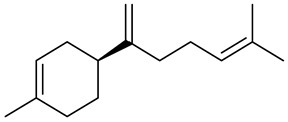
β-Sesquiphellandrene	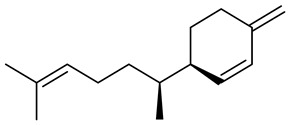
α-Panasinsen	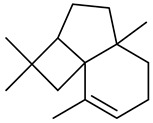
Germacrene B	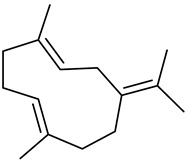
epi-Globulol	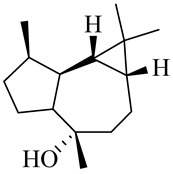
Cubenol	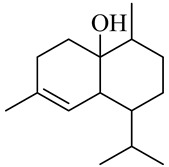
τ-Eudesmol	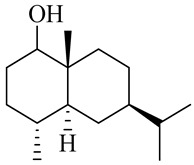
Caryophyllene oxide	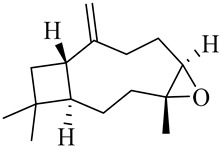
τ-Muurolol	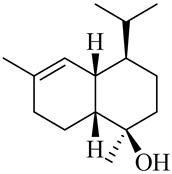
α-Cadinol	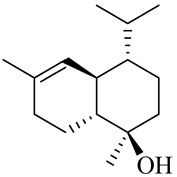
Spathulenol	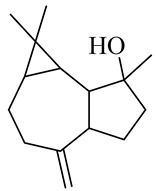
Cedr-8-ene	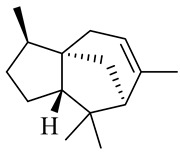
β-Cedren-9-α-ol	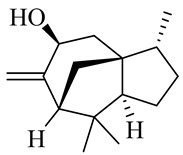
Farnesal	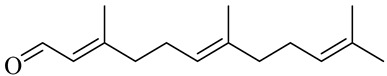
δ-Elemene	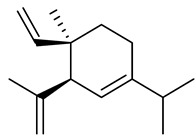
α-Caryophyllene	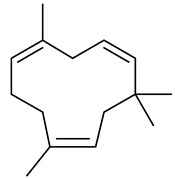
β-Caryophyllene	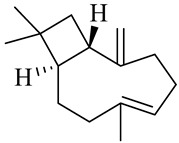
Bisabolene	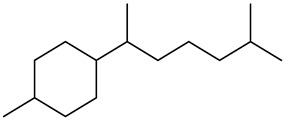
α-Humulene	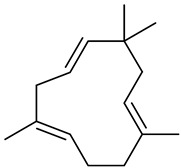
β-funebrene	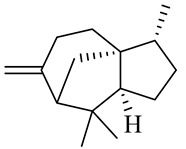
Sesquithujene	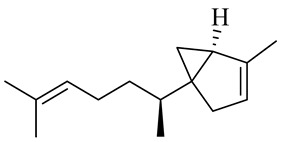
Calarene/Beta-gurjunene	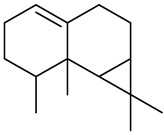
cis-nerolidol	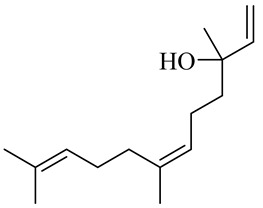
Elemol	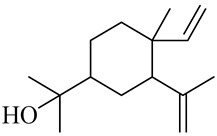
β-Atlantone	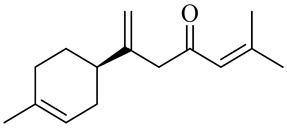
β-Eudesmol	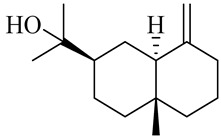
β-Bisabolol	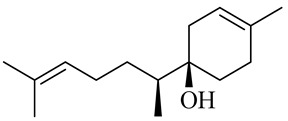
4-(1,5-Dimethylhex-4-enyl)cyclohex-2-enone	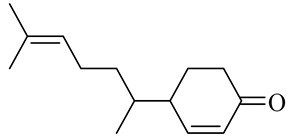
(2Z,6E)-Farnesol	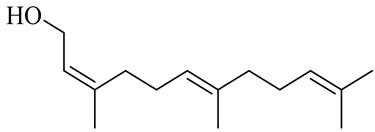
(2Z,6Z)-Farnesol	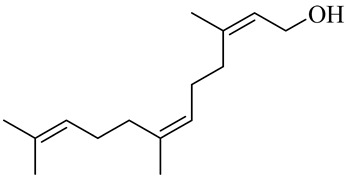
(2E,6E)-Farnesol	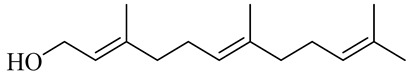
Isocaryophyllene	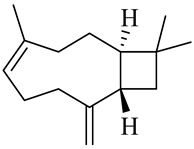
α-Humulene	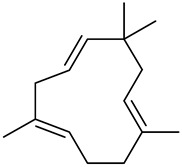
Germacrene D	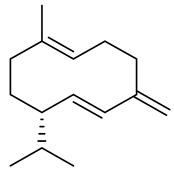
α-Selinene	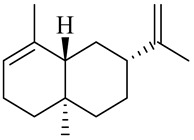
α-Muurolene	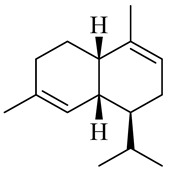
δ-Cadinene	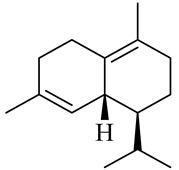
γ-Eudesmol	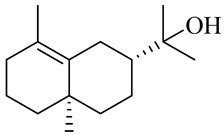
α-Bisabolol	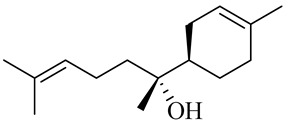
Caryophyllenol	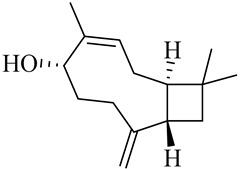
Diterpene	
Phytol	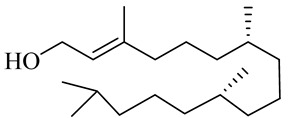
Flavonoids	
Rutin	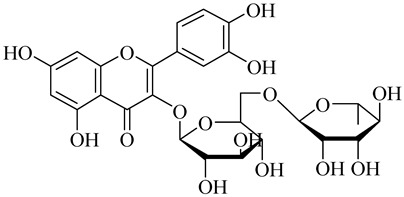
Apigenin	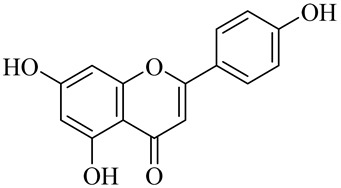
Myricetin	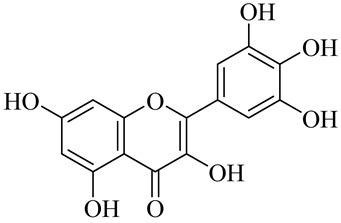
Naringenin	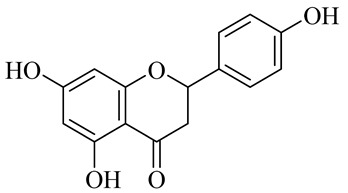
Fisetin	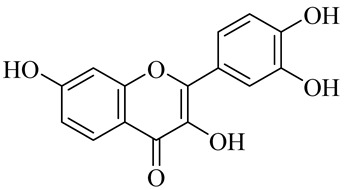
Morin	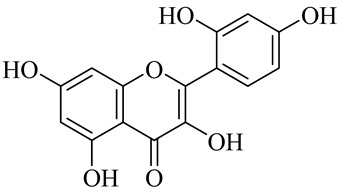
Flavylium/Anthocyanin	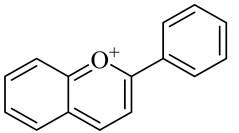
Quercetin	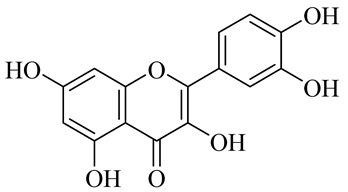
(−)-Epicatechin	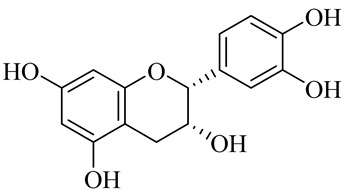
(+)- Catechin	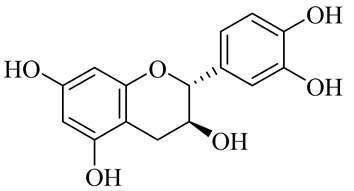
Kaempferol	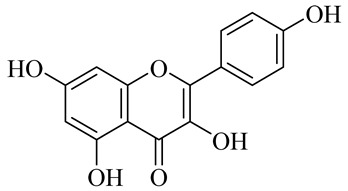
Naringenin	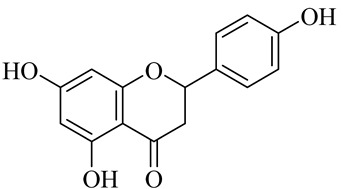
Organic acids	
Salicylic acid	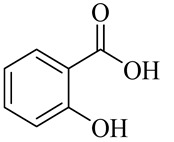
Cinnamic acid	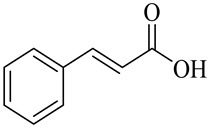
Gallic acid	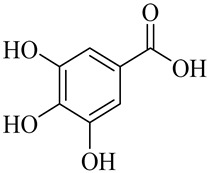
Vanillic acid	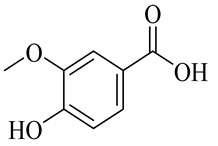
Ferulic acid	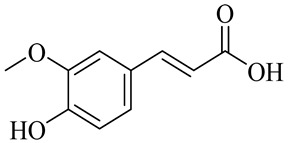
Tannic acid	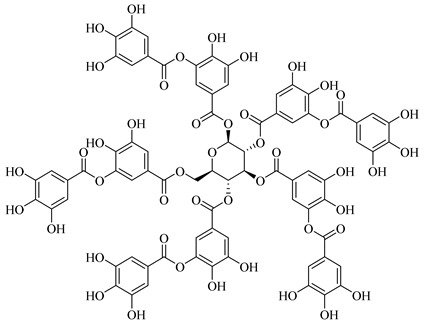
Caffeic acid	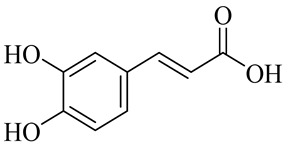
Ethyl cinnamate	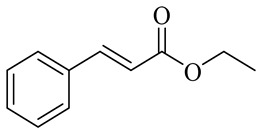
Ethyl p-methoxycinnamate	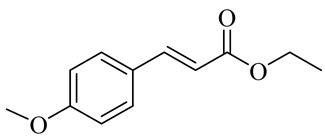
(2R,4S,5R)-2-(Acetoxymethyl)-6-(2-methoxy-4-((E)-3-oxodec-4-en-1-yl)phenoxy)tetrahydro-2H-pyran-3,4,5-triyl triacetate	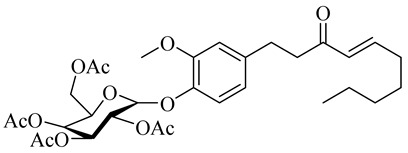
(E)-1-(3-Methoxy-4-(((3R,4S,6R)-3,4,5-trihydroxy-6-(hydroxymethyl)tetrahydro-2H-pyran-2-yl)oxy)phenyl)dec-4-en-3-one	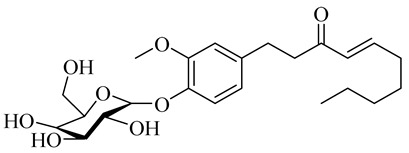
(2R,4S,5R)-2-(Acetoxymethyl)-6-(4-(5-hydroxy-3-oxodecyl)-2-methoxyphenoxy)tetrahydro-2H-pyran-3,4,5-triyl triacetate	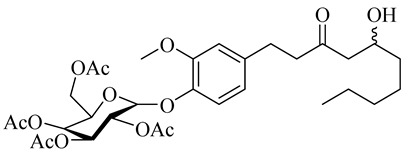
5-Hydroxy-1-(3-methoxy-4-(((3R,4S,6R)-3,4,5-trihydroxy-6-(hydroxymethyl)tetrahydro-2H-pyran-2-yl)oxy)phenyl)decan-3-one	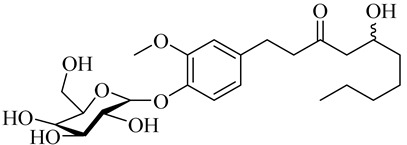
Amino acids	
Glutamine	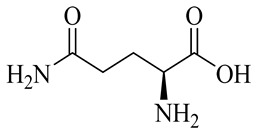
Histidine	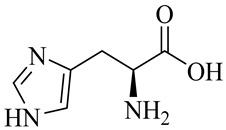
Glutamic acid	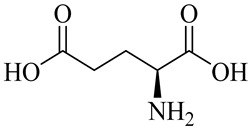
Threonine	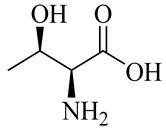
Leucine	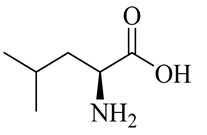
Lysine	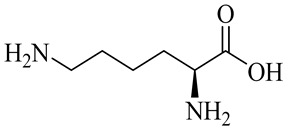
Valine	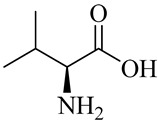
Tyrosine	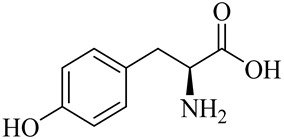
Miscellaneous	
2-Undecanone	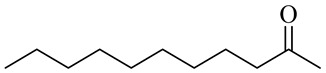
2-Heptanol	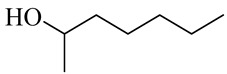
Octanal	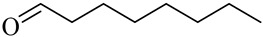
(E)-2-Octenal	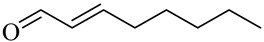
3,4-Dimethyl styrene	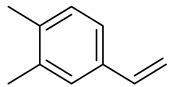
Naphthalene	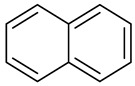
Cumene Isopropylbenzene	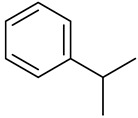
6-Hepten-3-one	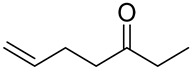
Naphthalene	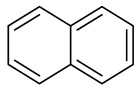
Tridecane	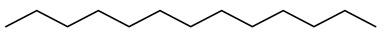
6-Methyl-5-hepten-2-oneSulcatone	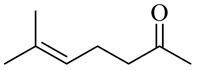
Nonanone	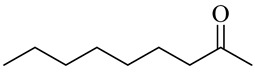
2-Heptyl acetate	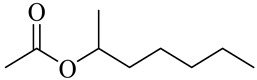
Trans-2-decenal	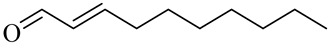

Note: The information in this table is obtained from [4,5,6,7,8,9,10,11,12,13].

**Table 2:** The biological activities and molecular mechanisms of red ginger.

Bioactivity	Mechanism	Responsible Constituents	Ref.
Antimicrobial activity	Anti-gram negative Anti-gram positive Natural preservative	β-caryophyllene	[33]
Analgesic activity	Hyperalgesia↓ γ-aminobutyric acid↓ NR2B↓	Camphene Cineole Geranial Geranyl acetate	[34,35,36]
Antidiabetic activity	α-amylase α-glucosidase	Gingerols shogaols	[37]
Anti-inflammatory	Prostaglandins↓ leukotrienes↓ COX-1↓ COX-2↓ 5-LO↓ TNF-α↓ NO↓ iNOS↓ NF-κb↓ TPA↓ ODC↓	6-gingerol 6-shogaol 8-gingerol 10-gingerol	[19,38,39,40,41,42]
Antioxidant	Donating electrons or a hydrogen atom to free radical	Phenolic compounds Quercetin 6-shogaol 8-shogaol 6-gingerol 8-gingerol 10-gingerol	[19,43,44,45]
Melanogenesis inhibitory activity	Melanogenesis inhibitory activity	Gingerdione	[25]
Anticancer and antitumor activity	Angiogenesis and metastasis	6-shogaol 6-gingerol	[46,47,48,49,50,51]
Antihyperlipidemic	LDL-C↓ ACE↓ HDL-C↓ malondialdehyde↓	Gingerol Shogaol Gingerdione	[52,53]
Anti-hypercholesterolemia	LDL↑, HMG-CoA↑, HDL↑	Phenolic compounds	[54]
Antihypertensive	NO↑ Prostacyclin Release Ca2+ Activation of cGMP-KATP Stimulation of muscarinic receptors	6-gingerol 6-shogaol gingerdione	[30]
Anti-Alzheimer’s disease	HDAC↓, Trichostatin A↓, MS275↓, AChE↑, α-secretase↓, Aβ-42↓, β-secretase↓, APH1a↑	Flavonoids Tannins Alkaloids 6-shogaol terpenoids	[20,53,55,56,57,58]
Androgenic effect	Pre-leptotene↑, pachytene spermatocytes↑	Arginine Shogaol Gingerdiol Gingerol Zingerone Zingiber	[59,60,61]
Insecticidal activity	Zingerone and benzaldehyde dimethyl thiol acetal	Zingerone Benzaldehyde dimethyl thiol acetal	[62]
Immunomodulatory	Recover small intestine mucosa structure of mice Protective effect on dopaminergic neurons	Phenols	[63]
